# Multicenter, 2-dose single-group controlled trial of tacrolimus for the severe infertility patients

**DOI:** 10.1097/MD.0000000000034317

**Published:** 2023-08-11

**Authors:** Michi Hisano, Koji Nakagawa, Masanori Ono, Osamu Yoshino, Takakazu Saito, Yasushi Hirota, Eisuke Inoue, Kayoko Kikuchi, Hidefumi Nakamura, Koushi Yamaguchi

**Affiliations:** a Center of Maternal-Fetal, Neonatal and Reproductive Medicine, National Center for Child Health and Development, Tokyo, Japan; b Center for Reproductive Medicine and Implantation Research, Sugiyama Clinic Shinjuku, Tokyo, Japan; c Department of Obstetrics and Gynecology, Tokyo Medical University, Tokyo, Japan; d Department of Obstetrics and Gynecology, Yamanashi University, Yamanashi, Japan; e Department of Obstetrics and Gynecology, The University of Tokyo, Tokyo, Japan; f Showa University Research Administration Center, Showa University, Tokyo, Japan; g Translational Research Headquarters, Fujita Health University, Aichi, Japan; h Department of Research and Development Supervision, National Center for Child Health and Development, Tokyo, Japan.

**Keywords:** infertility, maternal-to-fetal immunological abnormalities, tacrolimus

## Abstract

Infertility is estimated to affect 8% to 12% of reproductive-aged couples worldwide. While approximately 85% of infertile couples have an identified cause, the remaining 15% suffer physically and emotionally from unexplained intractable infertility. In recent years, maternal-to-fetal immunological abnormalities have attracted attention as mechanisms that differ from the conventional factors contributing to infertility and pregnancy loss. A T-helper 2 (Th2)-dominant immune state has been proposed as a maternal immune alteration to eliminate rejection and induce tolerance to a semi-allogeneic fetus. An imbalance in Th1 responses would not induce adequate maternal immune tolerance to the fetus or early embryos. Tacrolimus, widely used as an immunosuppressant agent in solid organ transplant recipients, is expected to suppress maternal rejection and promote tolerance to early embryos after assisted reproductive technology by modulating the immunological environment of the preimplantation endometrium. We planned an exploratory clinical trial to determine the efficacy, safety, and dosage of tacrolimus in women with intractable infertility.

**Methods and analysis:**

This is a multicenter, 2-dose, single-group controlled trial in infertile women who failed to achieve a chemical pregnancy despite multiple in vitro fertilization (IVF) and embryo transfer (ET) treatment cycles. The following 2 key selection criteria were set: no underlying factors of infertility despite appropriate evaluation and presence of Th1-dominant immune state, defined as a Th1/Th2 cell ratio ≥ 10.3 in the peripheral blood. A total of 26 eligible participants are randomly assigned (in a 2:1 ratio) to receive immunosuppressive therapy with oral tacrolimus at a daily dose of 2 mg or 4 mg. Tacrolimus is administered for 16 days starting from 2 days before ET. The primary endpoint is the presence of clinical pregnancy 3 weeks after IVF/ET treatment, and the secondary endpoint is the presence of biochemical pregnancy 2 weeks after IVF/ET treatment. Safety evaluation and biomarker discovery for tacrolimus treatment in infertile women will be conducted simultaneously.

**Trial registration number:**

Japan Registry of Clinical Trials (jRCT; jRCTs031220235).

## 1. Introduction

Infertility, a problem of the reproductive system defined as the failure to achieve a clinical pregnancy after 12 months or more of regular unprotected sexual intercourse,^[1]^ is estimated to affect 8% to 12% of reproductive-aged couples worldwide.^[2]^ Major causes of infertility can be categorized as ovulatory dysfunction, tubal occlusion, endometriosis, diminished ovarian reserve, uterine factors, and male factors.^[3]^ While approximately 85% of infertile couples have any of these identified factors,^[3]^ the remaining 15% suffer physically and emotionally from unexplained intractable infertility. In recent years, maternal-to-fetal immunological problems have been noted as a different mechanism from conventional factors contributing to infertility and pregnancy loss,^[4–6]^ but there are no appropriate evidence-based measures in clinical practice for the diagnosis and treatment of infertility that involve immune factors. During pregnancy, the maternal immune system is dramatically altered to eliminate rejection and induce tolerance to a semi-allogeneic fetus. A T-helper 2 (Th2) dominant immune state in the decidua has been previously proposed as one of the maternal immune alterations.^[7]^ In the peripheral blood, pregnant women with high Th1/Th2 ratios in the first trimester have been shown to shift towards Th2 dominance over the course of pregnancy.^[8]^ Assuming that cell-mediated immunity at the maternal-fetal interface is closely implicated in unexplained intractable infertility, a high Th1-dominant immune state would not induce adequate maternal immune tolerance to early embryos. This concept is similar to the rejection of solid organs after transplantation, and suppression of cellular immunity is considered reasonable for infertility treatment.

Tacrolimus, also known as FK506, was found as a 23-membered macrolide produced by *Streptomyces tsukubaensis*, which was isolated from a soil sample collected from Mount Tsukuba, Ibaraki, Japan, in 1984.^[9]^ The complex of FK506 and FK506 binding protein 12 negatively affects the cytoplasmic nuclear factor of the activated T cell pathway by blocking the activation of calcineurin, leading to suppression of cytokine expression such as interleukin 2. Calcineurin inhibitors are widely used as immunosuppressants in solid organ transplant recipients and patients with autoimmune disorders. Tacrolimus, in particular, has often been used for pregnant women after solid organ transplantation, and positive information on the safety of tacrolimus during pregnancy has been accumulated.^[10–13]^ These data suggest that maternal exposure to tacrolimus does not increase the incidence of congenital malformations or any recurrent pattern of malformations in offspring. The administration of tacrolimus for infertility involving immune factors is expected to reduce maternal rejection and promote tolerance to early embryos by modulating the immunological environment of the preimplantation endometrium. In 2015, we first reported on the efficacy of immunosuppressive treatment with tacrolimus in 42 infertile women with a peripheral blood Th1/Th2 ratio of ≥ 10.3, which resulted in a clinical pregnancy rate of 64.0% (16/25) in the treatment group compared with 0% in the control group (0/17).^[14]^ Furthermore, the proportion of peripheral blood Th1 cells was negatively correlated with clinical pregnancy rates following tacrolimus treatment in women with infertility repeated implantation failures.^[15]^ Tacrolimus could also have a potential efficacy against obstetrical complications associated with Th1 dominant immunity, such as recurrent pregnancy loss and preeclampsia.^[16,17]^

Tacrolimus is more commonly used for women with infertility in Japan, although it has not yet received regulatory approval for this purpose. The practical management for the use of tacrolimus is currently left at the discretion of individual fertility clinics and doctors. This exploratory clinical trial is conducted to determine the efficacy, safety, and dosage of tacrolimus treatment in women with intractable infertility who failed to achieve a chemical pregnancy after multiple embryo transfer (ET) cycles with morphologically good-quality embryos. The following 2 key selection criteria were set: no underlying factors of infertility despite appropriate evaluation and presence of Th1-dominant immune state, defined as a Th1/Th2 cell ratio ≥ 10.3 in the peripheral blood. In this study, tacrolimus will be administered for 16 days, starting from 2 days before ET, which corresponds to the period before fetal organogenesis.

## 2. Methods

### 2.1. Objective

This exploratory clinical trial aims to determine the efficacy, safety, and appropriate dosage of tacrolimus (Tacrolimus hydrate; Astellas Pharma Inc., Tokyo, Japan) in patients with intractable infertility.

### 2.2. Study design

**Figure 1.**
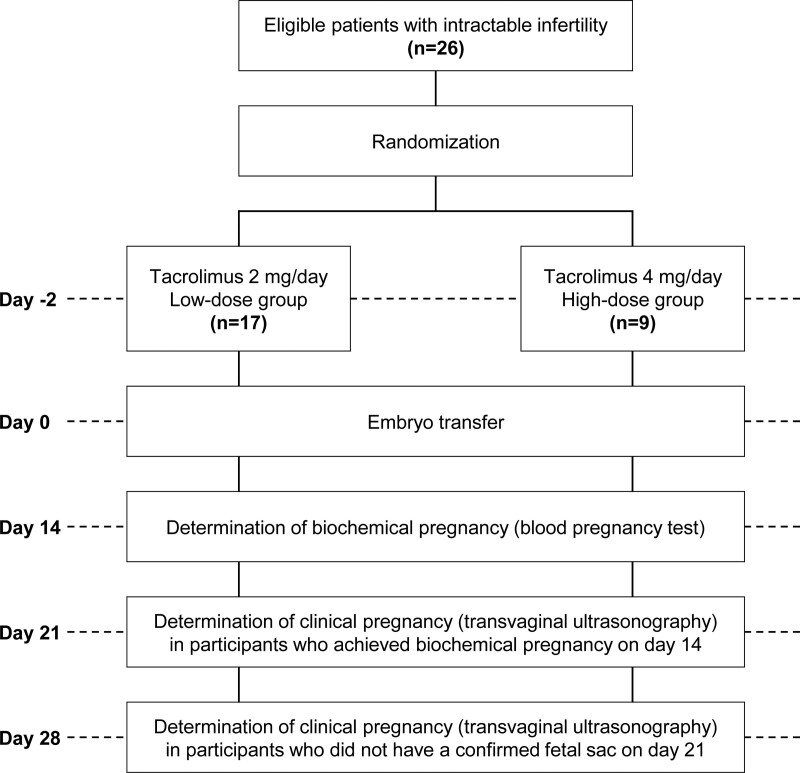
Flow of participants in this study.

This is a multicenter, 2-dose, single-group controlled trial of patients with intractable infertility (Fig. 1). Using an Electronic Data Capture system, eligible patients are randomly assigned 2:1 to receive either tacrolimus 2 mg once daily or tacrolimus 4 mg once daily. Oral administration of tacrolimus starts 2 days before ET and continues for a total of 16 days. The registration of subjects will be conducted at the following 4 facilities: National Center for Child Health and Development, Sugiyama Clinic Shinjuku, Tokyo Medical University Hospital, and University of Yamanashi Hospital.

### 2.3. Eligibility criteria

Study participants who meet all the following inclusion criteria and do not meet any of the exclusion criteria are eligible for the registration.

#### 2.3.1. Inclusion criteria.

1. Patients who have never had a biochemical pregnancy even after three or more ET cycles with morphologically good-quality embryos (regardless of fresh embryo, frozen embryo, early embryo, and blastocyst); a total of four or more embryos are used for ET. Ectopic pregnancies are not included in biochemical pregnancies
2. Patients in whom the cause of infertility has not been identified despite infertility evaluations including ovulatory dysfunction, tubal patency, uterine anatomic abnormalities, cervical factors, and quality and quantity of the partner’s sperm
3. Aged at least 18 years, but below 40 years at the time of registration
4. Th1/Th2 cell ratio ≥ 10.3 in the peripheral blood collected during the proliferative phase of the menstrual cycle
5. Patients with frozen embryos that are already stored for the purpose of assisted reproductive technology
6. Patients who can continue to visit or be hospitalized throughout the study period
7. Patients who are fully informed about the implementation of this study and have given their consent along with their partners in a document approved by the Clinical Research Review Board

#### 2.3.2. Exclusion criteria.

1. Patients who used immunosuppressive drugs for infertility treatment, such as tacrolimus, azathioprine, mizoribine, mycophenolate mofetil, cyclophosphamide, gusperimus, everolimus, ciclosporin, basiliximab, infliximab, etanercept, golimumab, tocilizumab, canakinumab, rituximab, abatacept, corticosteroids, and intravenous immunoglobulin
2. Patients diagnosed with chronic endometritis by endometrial histology
3. Patients infected with human immunodeficiency virus, hepatitis C virus, hepatitis B virus, or other active viruses; Patients under treatment or previously treated for infections caused by tuberculosis or invasive fungal infections; and those who are undergoing treatment for other bacterial infections
4. Patients with poorly controlled hypertension under antihypertensive drug treatment: systolic blood pressure ≥ 140 mm Hg/diastolic blood pressure ≥ 90 mm Hg
5. Patients with renal impairment: estimated glomerular filtration rate < 60 mL/min/1.73 m^2^
6. Patients with severe hepatic dysfunction: aspartate transaminase or alanine transaminase ≥2.5 times the upper reference limit for each facility
7. Patients with heart failure, ischemic heart disease, or serious arrhythmia and those with a history of these disorders
8. Patients with immunodeficiency diseases and those with a history of them
9. Patients with malignant tumors (including suspected patients) or a history of malignant tumors
10. Patients with a history of organ transplantation
11. Patients with a history of hypersensitivity to tacrolimus
12. Patients with the following blood abnormalities: white blood cell count < 2000/μL, neutrophil count < 1500/μL, and platelet count < 50,000/μL
13. Patients who participated in other interventional clinical trials within 6 months of registration or who plan to participate in other interventional clinical trials during this study period
14. Patients with untreated endocrine dysfunctions
15. Patients with blood coagulation abnormalities
16. Patients with antiphospholipid antibody syndrome and collagen vascular diseases associated with infertility or recurrent pregnancy loss
17. Patients or their partners with chromosomal aberrations
18. Patients receiving bosentan
19. Patients receiving potassium-sparing diuretics
20. Patients who receive live vaccines 2 months prior to the registration
21. Patients with other conditions that the principal investigator or sub-investigator deems ineligible

### 2.4. Intervention

#### 2.4.1. Treatment protocol.

Eligible severely infertile patients are randomly assigned (2:1 ratio) to receive immunosuppressive therapy with oral tacrolimus at a daily dose of 2 mg (low-dose group) or 4 mg (high-dose group). Randomization is based on the pre-allocated block sizes and stratified by the Th1/Th2 cell ratio (≥14.9 or <14.9) in the peripheral blood of the participants. The study drug is administered for a total of 16 days, starting 2 days before ET.

#### 2.4.2. Criteria for discontinuation of protocol treatment.

The principal investigator or sub-investigator will discontinue the intervention if any of the following events occur.

1. Participants request to discontinue the study drug intake.
2. Participants are unable to continue the therapeutic intervention due to adverse events and illness.
3. Unexpected administration of prohibited or restricted concomitant drugs to the participants.
4. Participants are found to have conflict with the eligibility criteria after the start of the protocol treatment.
5. Participants or their partners withdraw consent.
6. The principal investigator or sub-investigator determines that the subjects are unable to continue receiving the study drug.

#### 2.4.3. Prohibited concomitant drugs.

The following concomitant drugs and therapies are prohibited during the study period.

1. Drugs with immunosuppressive properties: tacrolimus excluding the study drug, azathioprine, mizoribine, mycophenolate mofetil, cyclophosphamide, gusperimus, everolimus, ciclosporin, basiliximab, infliximab, etanercept, golimumab, tocilizumab, canakinumab, rituximab, and abatacept.
2. Live vaccines.
3. Bosentan.
4. Potassium-sparing diuretics: spironolactone, potassium canrenoate, and triamterene.
5. Other investigational or unapproved domestic drugs.

### 2.5. Endpoints

#### 2.5.1. Primary endpoint.

The primary endpoint is the presence of clinical pregnancy 3 weeks after in vitro fertilization/ET treatment (V4). Clinical pregnancy is diagnosed by confirmation of the fetal sac through endovaginal ultrasonography. When the fetal sac cannot be confirmed, another ultrasound examination is performed 1 week later (V5). If the fetal sac is identified at that time, clinical pregnancy is diagnosed.

#### 2.5.2. Secondary endpoint.

The secondary endpoint is the presence of a biochemical pregnancy 2 weeks after in vitro fertilization/ET treatment (V3). Biochemical pregnancy is defined as a serum human chorionic gonadotropin (hCG) value ≥ 20 IU/L, but ectopic pregnancy is not included.

#### 2.5.3. Safety endpoints.

The following items are examined as safety endpoints.

- Number and percentage of participants with ectopic pregnancies (including suspected)
- Number and percentage of participants who developed any adverse event
- Number and percentage of each adverse event
- Number and percentage of adverse events by severity

### 2.6. Exploratory endpoints

Changes in the proportion and activity of effector CD4^+^ T cell subsets, such as Th1, Th2, Th17 and Th22 cells, regulatory CD4^+^ T cells, and NK cells in the peripheral blood are investigated both before and after therapeutic intervention. Using residual specimens from endometrial examination, biomarkers for the evaluation of endometrial receptivity and prediction of implantation failure will be explored at the University of Tokyo Hospital.

### 2.7. Study schedule

The schedule for informed consent, registration, tests, observations and assessments is shown in Table 1. The date are expressed with reference to the ET date (Date 0).

**Table 1:**
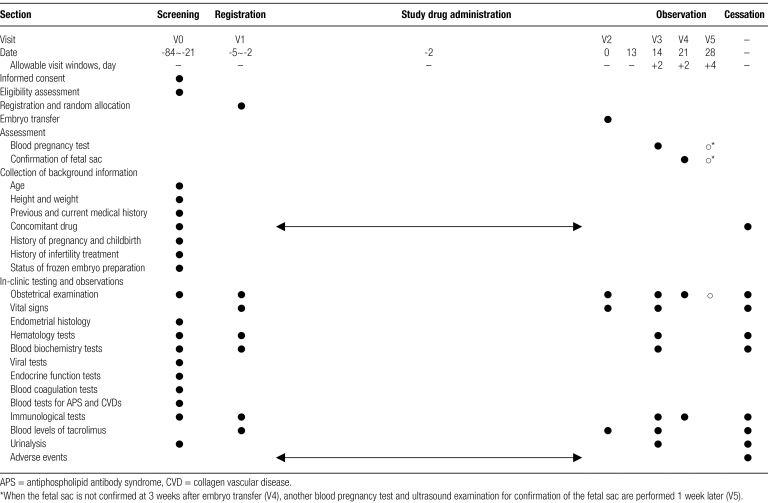
Study schedule.

#### 2.7.1. Informed consent (Date -84~-21).

At the commencement of the study, the principal investigator or sub-investigator obtains written informed consent from the participants and their partners to participate in this study, using an explanatory document prepared in accordance with the Clinical Trials Act and approved by the Clinical Research Review Board.

#### 2.7.2. Eligibility assessment (Date -84~-21).

Eligibility assessment is performed during the screening period.

#### 2.7.3. Registration and random allocation (Date -5~-2).

The principal investigator or sub-investigator confirms whether the subjects meet all the inclusion criteria and none of the exclusion criteria and performs the registration and random allocation of eligible subjects using the Electronic Data Capture (EDC) system (V1). The EDC system immediately checks patient and case registration information, issues a subject identification number, and assigns either a low- or a high-dose group if eligibility is met. The results of the assignment are automatically sent via e-mail to the principal investigator, sub-investigator, and the data center for registration confirmation notification. The principal investigator or sub-investigator will review the allocation results and perform the assigned study intervention.

#### 2.7.4. Assessments (Date 14, Date 21).

A blood pregnancy test will be performed on the last day of study drug administration (V3). If serum hCG levels are below 20 IU/L, that is, biochemical pregnancy is not confirmed, the study will be terminated. In participants, who achieved biochemical pregnancy, transvaginal ultrasonography for confirmation of the fetal sac will be performed 1 week after completion of the protocol treatment (V4). When the fetal sac cannot be confirmed, another blood pregnancy test and ultrasound examination to confirm the fetal sac will be performed 1 week later (V5). If serum hCG levels at this time are higher than that at 2 weeks after ET (V3), even though the fetal sac is not confirmed, the patient should be followed up for suspected ectopic pregnancies.

#### 2.7.5. Collection of background information.

For eligibility screening of the subjects, the principal investigator or sub-investigator collects the background information, including age, height, weight, history of previous and current medical illnesses, concomitant drugs, history of pregnancy and childbirth, history of infertility treatment, and status of frozen embryo preparation during the screening period. Concomitant drugs are checked at every visit to the clinic. The status of frozen embryo preparation includes the date and number of eggs retrieved, Veeck’s grades of early embryos, and Gardner’s grades of blastocysts. This information is documented in the electronic medical records.

#### 2.7.6. In-clinic testing and observations.

- Vital signs, such as body temperature, heart rate, and blood pressure are checked during registration, drug administration, and at study drug discontinuation.
- Histological examination of the endometrium (5–7 days after ovulation) is performed during the screening period to confirm that the participants do not have chronic endometritis. The histological diagnostic criterion for chronic endometritis is defined as the presence of five or more CD138-positive plasma cells in 10 visual fields in the endometrial stroma.
- The laboratory tests used in this study are listed in Table 2. Viral, endocrine function, blood coagulation, and antiphospholipid antibody syndrome and collagen vascular diseases tests are performed to identify eligible participants. Immunological tests are performed for the exploratory research.
- Blood levels of tacrolimus are measured during registration, drug administration, and study drug discontinuation.
- Information on adverse events occurring during the drug administration and observation period is collected. Details of adverse events are noted separately.

**Table 2:** Laboratory test items.

Classification	Items
Hematology tests	White blood cell count, hemoglobin, platelet count, fibrinogen, aPTT, FDP
Blood biochemistry tests	AST, ALT, total protein, albumin, BUN, creatinine, uric acid, zinc, CRP
Viral tests	HBs Ag, anti-HBs Ab, anti-HBc Ab, anti-HCV Ab, anti-HIV Ab
Endocrine function tests	FT4, TSH, prolactin, estradiol, LH, FSH, HbA1c
Blood coagulation tests	Factor XII Act, protein S Ag/Act, protein C Ag/Act, anti-thrombin Act
Tests for APS and CVDs	Lupus anticoagulant, anti-CL IgG/IgM Ab, anti-β2GP1 Ab, anti-PS/PT Ab, anti-PE IgG/IgM Ab, anti-nuclear Ab, anti-DNA Ab
Immunological tests	Proportion of CD4^+^, CD8^+^, IFN-γ^+^/CD4^+^, TNF-α^+^/CD4^+^, T-bet^+^/TNF-α^+^/CD4^+^, IL-4^+^/CD4^+^, Foxp3^+^/CD25^+^/CD4^+^, Foxp3^high^/CD25^+^/CD4^+^, IFN-γ^+^/IL-17A^+^/CD4^+^, and IL-17^-^/ IFN-γ^-^/IL-22^+^/CD4^+^, T cell, proportion of CD16^+^/CD56^+^/CD3^-^ NK cell, NK cell Act
Urinalysis	Sugar, protein, occult blood

β2GP1 = β2-glycoprotein-1, Ab = antibody, Act = activity, Ag = antigen, APS = antiphospholipid antibody syndrome, CL = cardiolipin, CVD = collagen vascular disease, PE = phosphatidylethanolamine, PS/PT = phosphatidylserine/prothrombin.

### 2.8. Adverse events

#### 2.8.1. Definition of adverse events.

Adverse events in this study are defined as any clinically untoward problem that develops in the participants from the date of protocol treatment initiation (V1) to the end of the observation period (V4). These events do not necessarily imply causal relationship with treatment interventions in this study.

#### 2.8.2. Definition of serious adverse events.

Serious adverse events refer to any of the following among the above events.

1. Death
2. Clinical conditions that might lead to death
3. Clinical conditions that require hospitalization for treatment or prolong the duration of hospitalization
4. Disability
5. Clinical conditions that might lead to disability
6. Serious medical conditions comparable to 1) to 5)
7. Congenital diseases or anomalies in later generations

#### 2.8.3. Severity assessment.

The principal investigator or sub-investigator records the description of adverse events, severity, date of onset, date of improvement, outcome, and causal relationship to the study drug on the EDC system. The severity of adverse events is assessed on the basis of Table 3.

**Table 3:** Severity of adverse events.

Grade	Severity	Medical necessity	Description
1	Mild	None	Mild or no symptoms
2	Moderate	Ambulatory treatment	Limitations other than personal care
3	Severe	Hospitalisation	Limitations in activities of daily living
4	Critical	Urgent treatment	Life-threatening conditions
5	Unsurvivable		Death

### 2.9. Calculation of sample size

According to the medical records of tacrolimus-treated infertile patients from 2018 to 2019 at the Sugiyama Clinic, the proportions of positive hCG results at daily doses of tacrolimus 2, 3, and 4 mg were 44.4% (12/27), 58.8% (20/34), and 57.1% (8/14), respectively. The estimated proportions in the high-dose group in this trial were calculated from the proportion of 58.3% (28/48) in the combined 3 and 4 mg group. Since the miscarriage rate in the period between hCG positivity and fetal sac confirmation was 10.3%,^[14]^ the probability of confirming the fetal sac in the low-dose and high-dose groups was conservatively set at 35% and 47%, respectively. In addition, the hCG positive rate in the untreated group was 0% (0/17) in a previous study examining the efficacy of tacrolimus.^[14]^ Therefore, the hCG positive rate in the untreated group was highly estimated at 5.6% (1/18) and the control value was set at 5%. On the 1-sided binomial test with a significance level.0125 comparing the low-dose and high-dose groups with a control value of 5%, the power exceeds.8 when the number of evaluable subjects is 15 and 8, respectively. Considering the exclusion of a few subjects, the sample size is set to 26.

### 2.10. Statistics

#### 2.10.1. Analysis set.

##### 2.10.1.1. Full analysis set

Full analysis set (FAS) was defined as all enrolled participants excluding:

1. those who never received this protocol treatment
2. those for whom no data have been available since the start of this protocol treatment
3. those who subsequently found to have breached the eligibility criteria

##### 2.10.1.2. Per protocol set

Participants in the FAS with no significant deviations from the research protocol will be included in the per protocol set (PPS).

##### 2.10.1.3. Safety analysis set

Safety analysis set will include all participants who received at least 1 dose of the study drug.

#### 2.10.2. Analysis method.

Analysis of the primary endpoints will be performed on the FAS, with a secondary analysis on the PPS. The proportion of clinical pregnancies and their 2-sided 95% confidence intervals calculated for the low-dose and high-dose groups, respectively, will be compared with a control value of.05 using the binomial test (1-tailed). The significant level for each comparison is set at .0125 and the study drug is considered effective when the *P* value is lower than this value. Missing data are treated as no clinical pregnancy at 3 weeks after ET. As a subgroup analysis, the above proportions and 2-sided 95% confidence intervals are calculated for each group with Th1/Th2 ratios below and above 14.9. Sensitivity analysis of the primary endpoints, with missing primary endpoints complemented by secondary endpoints, will also be performed. Missing secondary endpoints in this sensitivity analysis are treated as no chemical pregnancies at 2 weeks after ET.

Analysis of the secondary endpoints will be performed on the FAS, with a secondary analysis on the PPS. The proportion of chemical pregnancies and their 2-sided 95% confidence intervals are calculated for the low- and high-dose groups, respectively. Missing data are treated as no chemical pregnancies at 2 weeks after ET.

Safety analysis will be performed on the safety analysis set. The proportion of adverse events and miscarriages and their 2-sided 95% confidence intervals are calculated for the low- and high-dose groups, respectively.

#### 2.10.3. Interim analysis.

No interim analysis will be conducted in this study.

### 2.11. Ethics and dissemination

This research falls under the category of Specified Clinical Research under the Clinical Trials Act in Japan, and all researchers in this study will observe the Clinical Trials Act and conduct the research in accordance with the Declaration of Helsinki. This clinical research design was reviewed and approved by the Clinical Research Review Board of the National Center for Child Health and Development. The implementation plan was registered in the Japan Registry of Clinical Trials (https://jrct.niph.go.jp/) and submitted to the Minister of Health, Labour, and Welfare. The results and implementation plan of this research will be published in international peer-reviewed medical journals and Japan Registry of Clinical Trials (jRCTs031220235).

### 2.12. Monitoring and auditing

On-site monitoring will be conducted to ensure that the study is being conducted safely and in compliance with the protocol and that the data are collected correctly. In the auditing, the auditor, who is independent from the study group, will check not only the research implementation status but also whether the monitoring is being conducted properly and any problems identified by the monitoring are being resolved appropriately.

### 2.13. Study period

This study began in December 2022 and will end in October 2024.

## 3. Discussion

A large number of couples suffer from unexplained intractable infertility that does not fall into the major categories of ovulatory dysfunction, male factor infertility, and tubal disease. Although immunologic tests are rarely included in the commonly suggested evaluation of subfertility, reproductive failures, including unexplained infertility, recurrent pregnancy loss, and repeated implantation failure, are thought to be highly associated with maternal-to-fetal immunological problems. During pregnancy, various immune cells, extravillous trophoblasts, and decidual stromal cells form a dense network of cellular connections to balance immunity between the mother and fetus. It is equally important for the mothers to be immunologically ready to accept early embryos at the time of implantation. Immunotherapeutic approaches, including corticosteroids, calcineurin inhibitors, intravenous immunoglobulin, and anti-TNF agents, could be targeting the maternal immune system against early embryos or fetuses.^[4–6]^ In this clinical study, the calcineurin inhibitor tacrolimus will be administered to Th1-dominant women with intractable infertility. Th1-dominant immune status is defined as a Th1/Th2 cell ratio ≥ 10.3 in the peripheral blood, which was previously determined by adding 1 standard deviation to the mean Th1/Th2 cell ratio of 28 women with a history of normal delivery.^[14]^ This exploratory study will look at changes in the blood levels of Th1 and Th2 cells as well as other T-cell subsets and natural killer cells to assess the maternal immune status before and after the therapeutic intervention. These laboratory data would be useful in the search for diagnostic, prognostic, and monitoring biomarkers for tacrolimus treatment in patients with infertility. In addition, we are preparing to conduct an observational study to survey pregnancy outcomes and clinical information on mothers, fetuses, and neonates among the study participants who have achieved pregnancy on tacrolimus treatment. Some of these participants are still likely to remain at risk of rejecting the fetus because of their inability to maintain adequate immune tolerance during pregnancy. Tacrolimus can resolve various immunological problems between the mother and fetus, resulting not only in the treatment of immune factor infertility but also in the prevention of pregnancy complications, such as recurrent pregnancy loss and preeclampsia. Since the duration of tacrolimus treatment in this intervention study is restricted to 16 days before and after ET, it would be difficult to achieve the latter effect of preventing pregnancy complications. Therefore, it is important to continuously follow the pregnancy outcomes of the study participants until delivery.

The main limitation of this study is the lack of a placebo control group. We designed a 2-dose, single-group controlled trial after careful consideration of the ethical aspects of participants’ welfare. Another limitation is that it is difficult to explain infertility involving maternal-to-fetal immune abnormalities solely on the basis of the Th1/Th2 cell cytokine balance. Finally, the sample size is too small to detect rare adverse events when assessing the safety of tacrolimus treatment in patients with infertility.

## Acknowledgments

The authors thank Minami Shimada and Shoko Imai of the Clinical Research Center, National Center for Child Health and Development, Japan for their writing support to this protocol.

## Author contributions

**Investigation:** Koji Nakagawa, Masanori Ono, Osamu Yoshino, Takakazu Saito, Yasushi Hirota.

**Project administration:** Kayoko Kikuchi.

**Supervision:** Hidefumi Nakamura, Koushi Yamaguchi.

**Validation:** Eisuke Inoue.

**Writing – original draft:** Michi Hisano.

**Writing – review & editing:** Kayoko Kikuchi, Hidefumi Nakamura, Koushi Yamaguchi.
